# Real-World Data from the Use of Ranolazine in Patients with Stable Angina Pectoris: The RANGER Study

**DOI:** 10.3390/jcm13061672

**Published:** 2024-03-14

**Authors:** Christoforos Olympios, Panagiotis Stafylas, Alkiviadis Dermitzakis, Ioannis Efthimiadis, Alexandros Gardikiotis, Stavros Kakouros, Stylianos Lampropoulos, John Barbetseas, Angelos Sourgounis, on behalf of RANGER Investigators

**Affiliations:** 1 Cardiology Department, Thriassio General Hospital of Elefsina, 196 00 Magoula, Greece; 2 Healthink, Pylaia, 570 01 Thessaloniki, Greece; 3 Cardiology Department, Venizelio General Hospital, 714 09 Heraklion, Greece; 4 Bioclinic Thessaloniki, 546 22 Thessaloniki, Greece; 5 Cardiology Department, 417 NIMTS Veterans’ Fund Hospital of Athens, 115 21 Athens, Greece; 6 Cardiology Department, Sismanogleio General Hospital, 151 26 Athens, Greece; 7 Cardiology Department, Mamatseio General Hospital, 501 00 Kozani, Greece; 8 Cardiology Department, General Hospital “LAIKO”, 115 27 Athens, Greece; cardiologydept@laiko.gr; 9 Cardiology Department, 424 General Military Hospital, 564 29 Thessaloniki, Greece

**Keywords:** ranolazine, antianginal medicine, angina pectoris, coronary artery disease, real-world data

## Abstract

**Background**: Although ranolazine has been available for years as a second-line treatment to reduce angina attacks in patients with stable angina pectoris, real-world data on the effectiveness, tolerability, and safety of ranolazine are limited. **Methods**: A non-interventional, prospective study was conducted to assess the effectiveness and safety of ranolazine. Patients eligible for enrolment had a baseline assessment between one and fourteen days after initiating ranolazine for the first time and a follow-up visit three months later. The primary endpoints comprised the weekly frequency of angina attacks, total adverse events, and ranolazine discontinuation rate. The secondary endpoints included the use of short-acting nitrates, changes on the Canadian Cardiovascular Society (CCS) angina classification score and quality of life scale score (QoL). **Results**: In total, 1101 patients were enrolled at 214 sites. Mean weekly angina attacks were reduced from 3.6 ± 2.9 to 0.4 ± 0.9 (*p* < 0.0001) and the mean weekly consumption of short-acting nitrates decreased by 1.7 ± 2.2 (*p* < 0.0001). CCS class and QoL were also improved (*p* < 0.0001). Adverse events were reported by 11 (1%) patients in total, while 2 of them (0.2%) were characterised as serious. Treatment was discontinued for various reasons in 23 patients (2.1%) after the follow-up period. Ranolazine treatment was equally effective in all subgroups tested, with larger benefits observed in patients with more frequent angina and CCS angina class III and IV. Up-titration of ranolazine during the study improved the outcomes. **Conclusions**: Ranolazine was well tolerated and effectively reduced angina attacks, with simultaneous improvement of the CCS class and QoL score in patients with stable angina.

## 1. Introduction

Stable angina pectoris (AP) indicates recurring myocardial ischemia and occurs whenever myocardial oxygen demand exceeds oxygen supply [1]. This clinical presentation belongs to chronic coronary syndromes (CCS), which are characterised by functional alterations of coronary circulation, including not only obstructive coronary artery disease (CAD) but also endothelial and microvascular coronary dysfunction [2,3]. Angina significantly restricts patients’ physical activities and substantially affects their quality of life (QoL) [4]. The primary goal in managing CCS is to reduce AP and exercise-induced ischaemia, prevent cardiovascular events and improve symptoms, prognosis, and QoL through appropriate medications, interventions, and lifestyle modifications [2].

Guidelines recommend beta-blocker (BB) and/or calcium-channel blocker (CCB) treatments as first-line anti-ischaemic drug therapy [2,5]. When a first-line treatment is either contraindicated, poorly tolerated, or inadequate in controlling angina symptoms, second-line treatment should be considered [2]. Although the available evidence of the impact of this therapeutic strategy on morbidity or mortality is still weak, long-acting nitrates (LAN), ranolazine, trimetazidine, and, to a lesser extent, ivabradine and nicorandil may prove beneficial in combination with a BB or a CCB as first-line therapy, whereas no data are available for nicorandil [2]. Recent clinical guidelines suggest a four-step approach adapted to each patient’s characteristics and preferences [2]. For most patients, the initial therapeutic choice should be either a BB or CCB. If angina symptoms remain uncontrolled, the second step should be the administration of a combination of a BB and dihydropyridine CCB. Finally, if the condition still remains uncontrolled, a second-line treatment should be added, followed by a third second-line treatment option. However, in the case of a non-optimal heart rate (lower than 50 bpm or higher than 80 bpm), LV dysfunction, heart failure, or low blood pressure, a different treatment approach should be followed. For example, in the case of a low blood pressure, if a patient does not respond to or cannot tolerate a low dose of a BB or CCB, the next step is to switch to ivabradine, ranolazine, or trimetazidine, and the third step involves combining two second-line treatments [2]. More recently, Manolis at al. [6] suggested a more straightforward treatment algorithm for the management of angina according to haemodynamic variables (heart rate > or ≤ 60 bpm, systolic blood pressure < or ≥ 120 mmHg) and comorbidities. For example, a patient with heart rate ≤60 bpm and a systolic blood pressure <120 mmHg should receive ranolazine or trimetazidine, whereas the preferred initial treatment for a patient with diabetes mellitus is a vasodilating BB or ranolazine or trimetazidine.

Ranolazine selectively inhibits the late sodium current in cardiac cells and it is indicated in adults as add-on therapy for the symptomatic treatment of patients with stable angina pectoris who are inadequately controlled or intolerant to first-line antianginal therapies (such as beta-blockers and/or calcium antagonists) [2,7], significantly reducing recurrent ischaemia, angina frequency, and improving exercise tolerance [8,9,10,11]. In accordance with the treatment algorithm suggested by Manolis at al. [6], ranolazine is an appropriate treatment choice for all presented clinical scenarios (diabetes mellitus, microvascular angina, atrial fibrillation, heart failure with reduced ejection fraction, significant conduction abnormalities, and chronic obstructive pulmonary disease) and all haemodynamic groups [6].

Although clinical trials have provided evidence of clinical effectiveness, real-world data on the use of ranolazine are still limited. The objective of this study was to assess the effectiveness, safety, and tolerability of ranolazine in patients with stable AP in everyday clinical practice in Greece. Moreover, a subgroup analysis was performed to identify patient characteristics that are associated with better clinical outcomes.

## 2. Methods

### 2.1. Study Design

The RANGER study was a non-interventional, prospective, longitudinal, open-label, phase IV study of two visits assessing the effectiveness, safety, and tolerability of ranolazine among patients with stable AP in routine clinical practice at 214 investigational sites (8 hospitals and 206 private practice cardiologists) throughout Greece. The study was conducted between October 2015 and December 2017. The sites and patients were selected to represent the Greek population and clinical practice in Greece, where patients with stable CAD are managed mainly in the primary sector, whereas patients with cardiovascular events are mainly treated in the hospital sector. Each investigator had to enrol up to five adult patients suffering from stable AP and having recently initiated treatment with ranolazine. The study included a baseline assessment and a follow-up assessment three months later. The baseline assessment included: demographic and clinical characteristics, date and method of initial diagnosis of CAD, date and method of revascularisation, risk factors, self-reported stress/anxiety and physical inactivity, systolic and diastolic blood pressure and heart rate, number of angina attacks per week and use of short-acting nitrates per week, the Canadian Cardiovascular Society (CCS) angina classification, QoL score (investigator-assessed and self-reported with a 10-grade analogue scale, from 1 for no impairment to 10 for severe impairment in everyday life), cardiovascular and concomitant treatment, dosage, and reasons for initiating ranolazine treatment. The follow-up assessment included: the dosage of ranolazine treatment, cardiovascular and concomitant treatment, systolic and diastolic blood pressure and heart rate, number of angina attacks per week and use of short-acting nitrates per week, CCS classification, QoL, adverse events, rate, and reasons for discontinuation of ranolazine. The study was conducted following the Declaration of Helsinki and in agreement with the guidelines of the International Council for Harmonisation (ICH). The scientific councils of all participating hospitals approved the study protocol.

### 2.2. Participants

Eligible patients were adults suffering from AP who had recently initiated treatment with ranolazine (within a two-week period before enrolment) according to the Summary of Product Characteristics [7] and had signed an informed consent form. Exclusion criteria included hypersensitivity to the active substance or any of the excipients, simultaneous treatment with potent CYP3A4 inhibitors, severe renal dysfunction (creatinine clearance < 30 mL/min), moderate or severe liver dysfunction, any concomitant therapy with antiarrhythmic agents class Ia or III except amiodarone, pregnancy, or lactation, use of a greater than 1000 mg daily dose of metformin during the study, use of a greater than 20 mg daily dose of simvastatin, and prior treatment experience with ranolazine. Patients were free to withdraw from the study at any time and for any reason.

### 2.3. Outcomes Measurements

The primary effectiveness endpoint was the frequency of angina attacks per week, the primary safety endpoint was the type and frequency of adverse events (A.E.), and the primary tolerability endpoint was the discontinuation rate of ranolazine treatment. Secondary endpoints included the frequency of use of short-acting nitrates and the impact on CCS classification and QoL scores.

### 2.4. Statistical Analysis

The effectiveness and safety of ranolazine have already been assessed in large randomized clinical trials, where a reduction in the weekly rate of angina episodes due to ranolazine has been estimated to be in the range of 12–24% [7,12]. Under the assumptions of normally distributed data, a sample size of 1052 patients was projected to have 90% power to detect a reduction of a minimum of 10% in the primary endpoint [13]. After considering a potential 10% dropout rate, it was estimated that 1157 patients would need to be enrolled [13].

Continuous normal/skewed variables were summarised using mean/median, standard deviation/IQR, and range. The normality of the data was assessed using a normal quantile plot. Categorical variables were summarised using frequency and percentages. Baseline characteristics between males and females were compared using *t*-tests or chi-square tests. Absolute differences between the baseline and follow-up visit were evaluated by using the paired sample *t*-test or Wilcoxon test accounting for the nature of the data. The Pearson correlation coefficient was computed for the differences between baseline and follow-up visit of angina attacks and use of short-acting nitrates per week. An exploratory analysis was also performed between subgroups of the population assessing the effectiveness of ranolazine by the mean reduction in angina attacks and use of short-acting nitrates per week. All tests were two-sided, and the significance level was 0.05. Missing data were not imputed. Multiple linear regression (stepwise selection) was used to test the relationship of the difference in angina attacks between the baseline and follow-up visit, with the following independent variables: angina attacks and use of short-acting nitrates at baseline, gender, age, body mass index (BMI), CCS class at baseline, beta-blocker use, and SBP/DBP/HR difference between the baseline and follow-up visit. The data management and statistical analysis were performed using Stata, v16 (StataCorp LLC, College Station, TX, USA).

## 3. Results

### 3.1. Baseline Demographic and Clinical Characteristics

**Figure 1:**
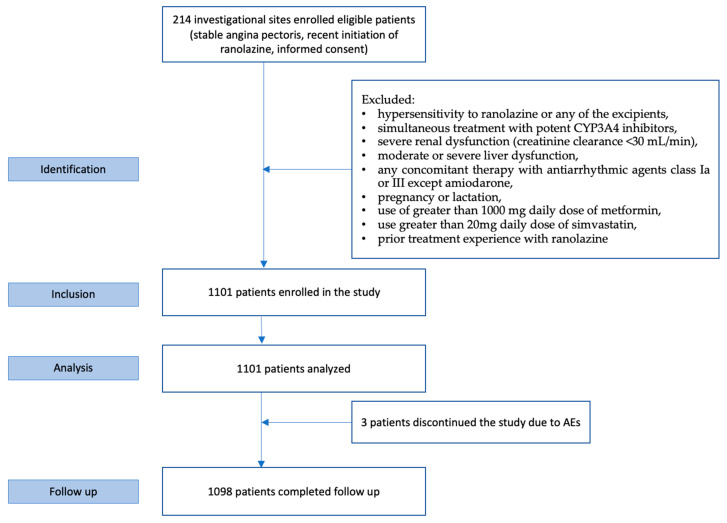
RANGER study patient flow chart.

In total, 1101 patients with stable AP and recent initiation of ranolazine were enrolled in the study (Figure 1). Three patients withdrew from the study before the follow-up visit due to adverse events and 1098 patients performed both the baseline and follow-up visits. The mean age of the enrolled patients was 71.3 ± 10.3 years (Table 1). Most of them were males (73.3%) and suffered from hypertension (76.6%) and hyperlipidaemia (75.5%); one third suffered from diabetes mellitus and one fourth from obesity. About 42.7% had at least three cardiovascular risk factors (CVRFs), and more than half of them reported a lack of physical activity and/or anxiety/stress. Despite AP, about one-fifth of the cohort were still smoking, and 34.3% were ex-smokers. The majority of males were younger (70.7 ± 10.1 vs. 73.4 ± 10.6, *p* = 0.022), smokers (*p* < 0.0001), and had a diagnosis of diabetes (*p* = 0.035) compared to women.

The median time from first angina symptoms was 6 months (IQR 0.2–3 years). Physical activity, stress, and cold weather were the leading causes that provoked symptoms in the patients (Table 2). According to the baseline medical history information, the diagnosis of AP was mainly clinical, based on symptom assessments (84.9%) and/or medical history (82%) and, in less than half of the patients, on coronary angiography (49.1%) or a stress test (42.0%). However, coronary angiography was ultimately performed in 792 patients (71.9%) and CAD was documented in 724 patients (91.4%). Revascularisation was performed in 337 patients (46.5%) and no revascularisation was reported in 358 (49.4%). Among the patients with documented CAD, percutaneous coronary intervention (PCI-with stent) was performed in 248 patients (34.3%), balloon angioplasty in 71 (9.8%), and coronary artery bypass grafting (CABG) in 124 (17.1%). A second revascularisation intervention was performed in 139 patients (19.2%).

Concerning baseline anti-ischaemic drugs, 83.2% were on beta-blockers, 40.8% were on CCBs, and 45.5% were on long-acting nitrates. Despite appropriate medical treatment, patients were symptomatic and ranolazine was initiated mainly to relieve angina (94.1%) and improve everyday life activities (89.3%) (Table S1).

### 3.2. Ranolazine Exposure and Safety

Most patients (87.5%) started with the recommended dose of 375 mg ranolazine twice daily, whereas 106 (9.6%) of them initiated their treatment with a 500 mg dose of ranolazine twice daily and just 3 (0.3%) patients started with a 750 mg dose twice daily based on their physician’s judgment (Table S2). The dose was increased in approximately half of the participants (50.3%) before the follow-up visit, whereas for 46.9% of the patients the dose remained stable. For two of the three cases initially received a dosage of 750 mg twice daily (bid), the dosage was reduced to 500 mg, bid.

During the three months of the follow-up period, among 1101 patients only 11 (1%), all aged 70 years or older, reported a total of 21 AEs. Two of these patients reported serious AEs (a case of “not-related to the treatment” pulmonary embolism, where the patient died, and a case of second degree atrioventricular block that was resolved) (Table S3). The most frequently reported AEs were dizziness (n = 4 patients) and nausea (n = 3 patients). No adverse events were reported for patients with heart failure or mild-to-moderate kidney disease. In 23 cases (2.1%), ranolazine treatment was discontinued after the follow-up period, primarily due to adverse events (0.7%), patient decisions (0.6%), and no improvement of symptoms (0.4%).

### 3.3. Treatment Outcomes

**Figure 2:**
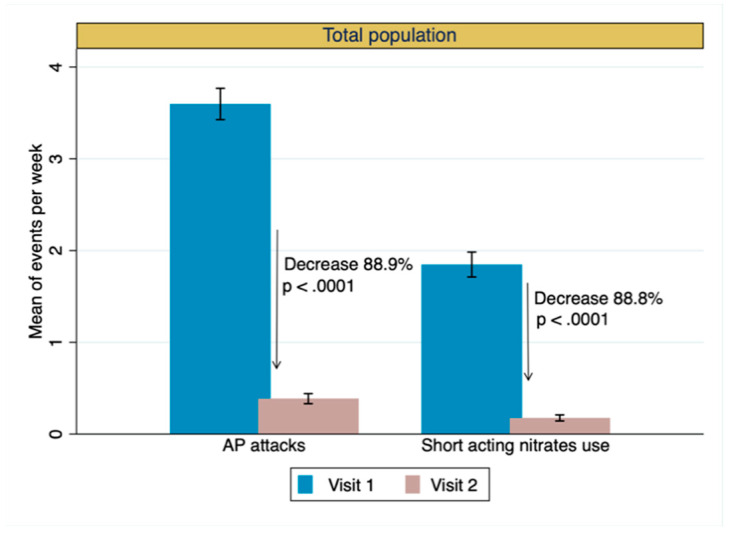
Mean (95%CI) number of angina pectoris (AP) events and use of short-acting nitrates per week in the course of both visits by total population.

**Figure 3:**
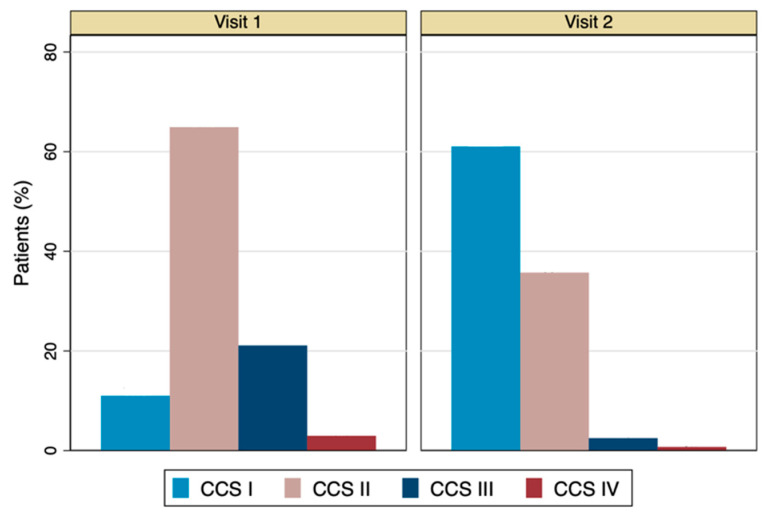
CCS grading throughout the course of the two visits. CCS scale: CCSI, No angina during normal activity; CCSΙI, Light limitation of ordinary activity; CCSIII, Considerable limitation of ordinary physical activity; CCSIV, Inability to carry on any physical activity without discomfort, angina syndrome maybe presents at rest.

**Figure 4:**
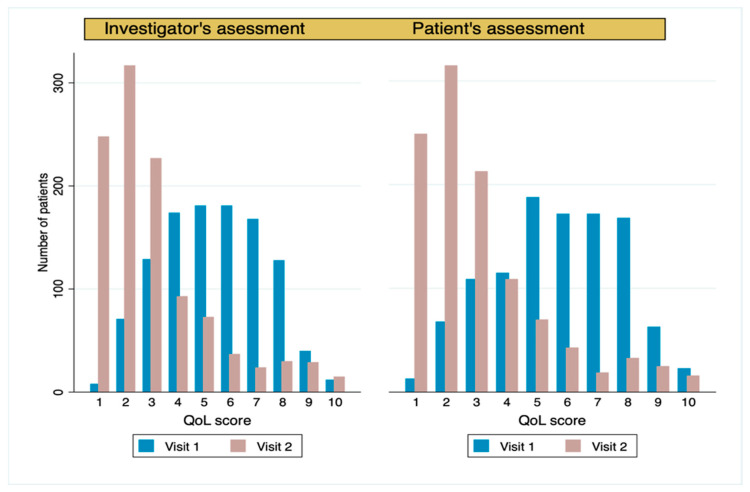
Quality of life (QoL) at both visits classified by investigators and patients. QoL scale 1–10, where 1: no impairment in everyday life to 10: severest impairment in everyday life.

The average weekly frequency of angina attacks (primary effectiveness endpoint) was significantly reduced from 3.6 ± 2.9 at baseline to 0.4 ± 0.9 at the end of the study (absolute difference −3.2 ± 2.7; *p* < 0.0001) (Figure 2, Table 3). The mean weekly use of short-acting nitrates decreased from 1.8 ± 2.3 to 0.2 ± 0.5 (absolute difference 1.7 ± 2.2; *p* < 0.0001). The reduction in angina attacks was positively correlated with the reduction in the use of short-acting nitrates (r = 0.6, *p* < 0.0001) (Figure S1). A significant improvement in CCS class (Wilcoxon signed-rank; *p* < 0.0001) (Figure 3, Table 3) was observed after three months of ranolazine treatment. CCS angina class was improved in 703 (63.9%) patients, remained stable in 365 (33.2%), and worsened in 30 (2.7%) (Tables S4 and S5). A significant improvement in the quality of life was shown after three months of ranolazine treatment (Figure 4, Table S5). The investigators’ and patients’ evaluations of QoL were consistent, demonstrated by the strong positive correlation that was observed (r = 0.84, n = 1098, *p* < 0.0001).

Consistent with the improvement in QoL between the two study visits, 74,2% of patients had no more angina symptoms at the end of the study. Analogously, 91,3% of the patients reported increased endurance during everyday activities and 93,8% relief from angina symptoms.

Exploratory sub-group analyses showed that ranolazine treatment was equally effective in both genders, patients of younger and older ages, diabetics and non-diabetics, hypertensives, and non-hypertensives, the obese and non-obese, and patients with and without a recent PCI or CABG (*p* > 0.05) (Table 4). Although most of the patients were significantly improved, subgroup analyses showed that patients with more than three angina attacks per week and those receiving more than two short-acting nitrates per week showed even better clinical outcomes (*p* < 0.001). Moreover, patients in CCS class III or IV had larger improvements than patients in CCS class I or II (*p* < 0.0001). Another interesting finding was that patients with ranolazine up-titration during the follow-up had even better outcomes than the patients without up-titration of ranolazine during the study.

Regression analysis (Table 5) also showed that patients with a higher number of angina attacks and an increased use of short-acting nitrates per week at baseline experienced greater improvements than the others. Regression analysis did not identify any other demographic characteristic that significantly correlated with the study’s primary outcome.

## 4. Discussion

The study presents the demographic and clinical characteristics of a representative sample of the Greek population suffering from stable AP and having recently initiated treatment with ranolazine, as well as the effectiveness, tolerability, and safety of ranolazine in this population. Recruiting 1101 patients at 214 investigational sites, RANGER is one of the biggest phase IV studies of ranolazine, allowing the collection of real-world data concerning the management of AP with ranolazine, which are notably limited. This is particularly interesting for settings like Greece, where such registries are not available.

The study showed that patients treated with ranolazine were usually males over 65 years old suffering from hypertension and hyperlipidaemia, and one-third of them were diabetics. Usually, they had multiple cardiovascular risk factors (>2) and an unhealthy lifestyle, including smoking, a lack of physical activity, obesity, and stress/anxiety. These findings can be directly compared with the results of the OSCAR-GR study published five years ago and including 189 patients from 20 centres [12]. Although the age, gender, BMI, and revascularisation metrics were similar, the frequency of cardiovascular risk factors and comorbidities were much higher in RANGER, e.g., frequency of diabetes 32.9% vs. 10% and smoking 16.4% vs. 13.8%. Patients reported a diagnosis of angina pectoris up to 42 years prior to inclusion, although most of them reported the first appearance of the symptoms during the year before enrolment. RANGER further confirmed that the diagnosis of AP remained clinical, with coronary angiography and exercise ECG being utilised less frequently. However, coronary angiography was performed in 71.9% of enrolled patients and, although CAD was documented in 91.4% of them, revascularisation treatment was performed only in 46.5% of them, which is similar to the results of other studies [12,14].

The effectiveness and safety of ranolazine have already been assessed and confirmed in randomised clinical trials (RCTs) [12,13,15,16]. The present study adds to the existing body of knowledge of the real-world effectiveness, safety, and tolerance of ranolazine, an essential adjunct to RCTs because they add data from daily-life clinical practice in specific settings and patients with comorbidities that usually are not included in RCTs [17].

One of the biggest contributions of the RANGER study to the literature is its insights into the safety and effectiveness of ranolazine in older patients with AP. A previous analysis of pooled data from two large, randomized trials with 363 patients aged 70 years or older showed that the efficacy of ranolazine was similar in older and younger patients but adverse events were more common in the elderly group [18]. The RANGER study, with 616 patients aged 70 years old or more and 247 patients aged 80 years old or more, confirmed this finding with data obtained from a real-world setting. Considering that advanced age is associated with frailty and adverse outcomes, treatment must be individualised considering the comorbidities, risk factors, and haemodynamic variables [19].

Treatment with ranolazine significantly reduced weekly angina attacks and the mean weekly consumption of short-acting nitrates, which was consistent with similar studies [13,14,16,20]. A significant improvement in CCS class and QoL was also found, which has already been demonstrated in other studies [12,14,20,21,22]. However, our study additionally indicates that although ranolazine treatment was effective in all subgroups tested, patients with a higher number of angina attacks per week and those with CCS angina class III or IV had the greatest improvements. Moreover, it was interesting to confirm that up-titration of ranolazine during the study significantly improved the outcomes.

Differences in vital signs were not considered clinically significant since it is known that ranolazine’s mechanism of action does not affect haemodynamic parameters [23], explaining why ranolazine is well tolerated by patients with low blood pressure and heart rate [2,11,24]. The statistical significance of vital signs may stem from the awareness of patients and physicians that they participated in the study and due to the fact that the study protocol allowed some changes in medications to better control these signs. Moreover, the study had a significant statistical power provided by the big sample size.

The low percentage of discontinuation and AEs confirmed the tolerability of ranolazine and was consistent with the already published safety results [11,12,14,20,23]. This study reports a lower number of AEs in comparison with another one from Greece [14]; however, the shorter follow-up period could be an explanation for the difference.

This study has some limitations that should be considered when interpreting the findings. The open-label, non-interventional study design might lead to a bias overestimating the actual effect of ranolazine and underestimating AEs; however, this allowed evaluating the drug effect under routine clinical practice. The follow-up period was too short to assess the incidence of major cardiovascular events and planned or unplanned cardiac interventions, and consequently, these variables have not been assessed in the follow-up visit and not been introduced in the multiple regression models. The patients were also receiving multiple medications and physicians were free to make any change to better control the symptoms and risk factors, rendering the evaluation of the effect of ranolazine more challenging. However, most of the patients had no change in their medications during the study. QoL was assessed using a 10-grade analogue scale and not with a validated generic (e.g., EQ5D) [25] or disease-specific questionnaire (Seattle Angina Questionnaire, SAQ) [26].

The RANGER study has also highlighted significant gaps in evidence, starting from the diagnostic strategy to the pharmacological management and revascularisation choices. We are still missing convincing evidence about the impact of the available anti-ischaemic medicines and their combination on the morbidity and mortality of the target population, especially for some special subpopulations, such as women, older or frail patients, etc. Moreover, neither the four-step approach recommended by the ESC nor other strategies suggesting the initial use of a second-line anti-ischaemic drugs either alone or in combination with a BB or a CCB have yet provided adequate evidence regarding the most effective treatment strategy. A combination of well-designed and adequately powered studies (both RCTs and RWD studies) is required to address these important clinical questions.

## 5. Conclusions

In conclusion, the RANGER study showed that, among patients suffering from stable AP who had recently initiated ranolazine, ranolazine was well tolerated and associated with fewer angina attacks and improved CCS class and QoL. Moreover, the study showed that patients with a higher number of angina attacks per week and in CCS angina class III or IV experienced larger improvements. Finally, yet importantly, up-titration of ranolazine during the study significantly improved the outcomes.

## Figures and Tables

**Table 1:** Baseline demographics and clinical characteristics of the study population.

Parameter	Total (N = 1101)	Female (N = 284)	Male (N = 807)	*p*-Value *
	n (%)	n (%)	n (%)	
**Age (y) (mean ± SD, range)**	71.3 ± 10.3 (39–93)	73.4 ± 10.6 (39–93)	70.7 ± 10.1 (40–93)	0.022
**Gender**		284 (25.8)	807 (73.3)	
**Body mass index (kg/** $m^{2}$ **)**				0.006
Mean ± SD, range	28.0 ± 3.7, (18.0–50)	27.9 ± 4.3, (18.9–43)	28.1 ± 3.5, (19.8–44)	
Normal (18.5–25)	204 (18.5)	64 (22.5)	139 (17.2)	
Overweight (25.1–29.9)	605 (54.9)	140 (49.3)	460 (57.0)	
Obese (≥30)	278 (25.2)	78 (25.3)	202 (25.0)	
**Smoking status**				<0.0001
Current smoker	181 (16.4)	20 (7.0)	160 (19.8)	
Ex-smoker	378 (34.3)	27 (9.5)	350 (43.4)	
Never smoker	419 (38.1)	203 (71.5)	211 (26.1)	
**Number of cardiovascular risk factors §**				0.042
0	85 (7.7)	29 (10.2)	53 (6.6)	
1	118 (10.7)	30 (10.6)	88 (10.9)	
2	367 (33.3)	95 (33.5)	277 (34.3)	
3	277 (25.2)	63 (22.3)	214 (26.5)	
4	163 (14.8)	43 (15.2)	120 (14.9)	
5	30 (2.7)	9 (3.2)	21 (2.6)	
**Comorbidities**				
Hypertension	843 (76.6)	216 (76.1)	621 (76.9)	0.103
Hyperlipidaemia	831 (75.5)	204 (71.8)	621 (76.9)	0.032
Diabetes mellitus	362 (32.9)	80 (28.2)	279 (34.6)	0.035
Parental CAD history	290 (26.3)	77 (27.1)	212 (26.3)	0.115
Physical inactivity	656 (59.6)	179 (63.0)	471 (58.4)	0.013
Stress/Anxiety	579 (52.6)	169 (59.5)	405 (50.2)	<0.0001
Cardiac arrhythmia	151 (13.7)	36 (12.7)	113 (14.0)	0.781
Heart failure	105 (9.5)	17 (6.0)	87 (10.8)	0.031
Hyperuricemia	96 (8.7)	15 (5.3)	79 (9.8)	0.037
Mild to moderate CKD	56 (5.1)	4 (1.4)	52 (6.4)	0.005
Depression	48 (4.4)	17 (6.0)	31 (3.8)	0.041
Other	82 (7.4)	17 (6.0)	63 (7.9)	0.319

§ The assessed cardiovascular risk factors included the following: hypertension, hyperlipidaemia, diabetes mellitus, parental CAD history, smoking (current), obesity, physical inactivity, stress/anxiety. Abbreviations: CAD—coronary artery disease; CKD—chronic kidney disease. * The *p*-values refer to the comparison between males and females using either a *t*-test or chi-square test.

**Table 2:** Symptoms, diagnosis, and management of CAD.

Parameter	Total (N = 1101)	Female (N = 284)	Male (N = 807)
	n (%)	n (%)	n (%)
**Time since first angina symptoms, y (median, IQR)**	0.5 (0.2–3.0)	0.5 (0.1–2.5)	0.6 (0.2–3.3)
≤1	634 (57.6)	172 (60.6)	455 (56.4)
1–3	174 (15.8)	49 (17.3)	123 (15.2)
>3	270 (24.5)	62 (21.8)	207 (25.7)
**Main causes that provoke symptoms in the patients**			
Physical activity	1017 (92.3)	255 (89.8)	752 (93.2)
Stress	677 (61.5)	202 (71.1)	468 (57.9)
Cold weather	427 (38.8)	106 (37.3)	318 (39.4)
Meal	270 (24.5)	64 (22.7)	204 (25.3)
Weather changes	214 (19.4)	51 (17.9)	160 (19.8)
Smoking/alcohol	71 (6.4)	13 (4.6)	58 (7.1)
Other	18 (1.8)	8 (2.5)	1 (1.2)
**Procedures used for angina diagnosis**			
Typical symptoms	936 (84.9)	251 (88.4)	676 (83.8)
Medical history	903 (82.0)	229 (80.6)	664 (82.3)
Coronary angiography	541 (49.1)	99 (34.9)	439 (54.4)
Stress test	463 (42.0)	93 (32.8)	365 (45.2)
Echocardiography	414 (37.6)	120 (42.3)	287 (35.5)
Scintigraphy	406 (36.9)	97 (34.2)	305 (37.8)
Stress echo	51 (4.6)	13 (4.6)	37 (4.6)
Other	22 (2.0)	7 (2.4)	15 (1.8)
**Coronary angiography**			
Total performed	792 (71.9)	182 (64.1)	600 (74.3)
Documented CAD	724 (91.4)	155 (85.2)	561 (93.5)
Revascularisation treatment *			
No	358 (49.4)	90 (58.1)	263 (46.9)
Yes	337 (46.5)	53 (34.2)	281 (50.1)
2nd revascularisation	139 (19.2)	25 (16.1)	112 (20.0)
Revascularisation methods (n. of patients) *			
PCI (w. stent)	248 (34.3)	44 (28.4)	201 (35.8)
CABG	124 (17.1)	15 (9.7)	108 (19.3)
Balloon angioplasty	71 (9.8)	13 (8.4)	58 (10.3)
**Pharmacological management**			
Antiplatelet	899 (81.8)	211 (75.0)	681 (84.4)
Beta-blockers	916 (83.2)	222 (78.2)	686 (85.0)
CCBs	449 (40.8)	121 (42.6)	323 (40.0)
Long-acting nitrates	501 (45.5)	108 (38.0)	387 (47.9)
Statins	520 (47.2)	119 (41.9)	400 (49.8)
ARBs	460 (41.8)	127 (44.4)	331 (41.0)
ACE inhibitors	364 (33.1)	78 (27.5)	282 (34.9)
Short-acting nitrates	287 (26.1)	73 (25.7)	212 (26.3)
Anticoagulants	63 (5.7)	15 (5.3)	48 (6.0)
Ivabradine	9 (0.8)	2 (0.7)	6 (0.7)
Other	415 (37.7)	88 (30.9)	325 (40.3)

* Among patients with documented CAD. Abbreviations: CAD—coronary artery disease; PCI—percutaneous coronary intervention; CABG—coronary artery bypass grafting.

**Table 3:** Treatment outcomes and vital signs.

Outcomes	Baseline	Follow-Up	Absolute Difference	*p*-Value
	(N = 1101)	(N = 1098)		
**Angina attacks (per week), mean ± SD**	3.6 ± 2.9	0.4 ± 0.9	−3.2 ± 2.7	<0.0001
**Use of short-acting nitrates (per week), mean ± SD**	1.8 ± 2.3	0.2 ± 0.5	−1.7 ± 2.2	<0.0001
**CCS angina classification**				<0.0001
CCS class I	122 (11.1%)	670 (60.8%)		
CCS class II	714 (64.8%)	392 (35.6%)		
CCS class III	230 (20.9%)	28 (2.5%)		
CCS class IV	32 (2.9%)	8 (0.7%)		
Quality of life *, mean ± SD				
Investigator’s assessment	5.5 ± 2	3 ± 2.1	−2.5 ± 2.4	<0.0001
Self-reported	5.7 ± 2.1	3.1 ± 2.1	−2.6 ± 2.6	<0.0001
**Self-reported improvement in tolerance of dailies activities**				
YES	-	1005 (91.3%)	-	
NO	-	61 (5.5%)	-	
**Self-reported symptoms relief**				
YES	-	1033 (93.8%)	-	
NO	-	47 (4.3%)	-	
**Vital signs**				
Systolic blood pressure (mmHg)	135 ± 15	127 ± 11	−8 ± 14	<0.0001
Diastolic blood pressure (mmHg)	80 ± 10	77 ± 8	−3 ± 9	<0.0001
Heart rate (bpm)	70 ± 10	67 ± 7	−3 ± 9	<0.0001

* QoL assessed with a 10-grade analogue scale: from 1—No impairment in everyday life to 10—severe impairment in everyday life. Abbreviations: CCS—Canadian Cardiovascular Society (CCS) angina class; SD—standard deviation.

**Table 4:** Treatment outcomes in various subgroup exploratory analyses assessed by mean reduction (∆) in angina attacks and use of short-acting nitrates per week.

Subgroups	∆ Angina Attacks	*p*-Value	∆ Use of Short-Acting Nitrates	*p*-Value
Male vs. female	−3.2 vs. −3.3	0.4114	−1.7 vs. −1.6	0.6472
Age, <70 vs. ≥70 yrs	−3.1 vs. −3.2	0.5873	−1.6 vs. −1.8	0.1182
Documented CAD with CA, yes vs. no	−3.1 vs. −3.4	0.4104	−1.7 vs. −0.8	**<0.0001**
Angina diagnosis ≤1 vs. >1 yrs	−3.3 vs. −3.1	0.6287	−1.7 vs. 1.7	0.6528
PCI, yes vs. no	−3.1 vs. −3.3	0.6443	−1.7 vs. −1.9	0.3464
CABG, yes vs. no	−2.9 vs. −3.2	0.5623	−1.5 vs. −1.8	0.4041
Angina attacks per week, ≤3 vs. >3 (baseline)	−1.8 vs. −5.7	**<0.0001**	−1 vs. −2.9	**<0.0001**
Short-acting nitrates per week, ≤2 vs. >2 (baseline)	−2.5 vs. −5.0	**<0.0001**	−0.6 vs. −4.4	**<0.0001**
CCS I-II vs. III-IV, (baseline)	−2.8 vs. −4.5	**<0.0001**	−1.4 vs. −2.6	**<0.0001**
Risk factors, yes vs. no	−3.3 vs. 2.8	**0.0335**	−1.7 vs. −1.6	0.6457
Diabetes, yes vs. no	−3.4 vs. −3.2	0.4574	−1.9 vs. 1.6	0.0784
Hypertension, yes vs. no	−3.3 vs. −3.2	0.7713	−1.7 vs. −1.4	0.0884
Cardiac arrythmia, yes vs. no	−3.2 vs. −3.6	0.2471	−1.7 vs. −2.0	0.2622
BMI, ≤30 vs. >30	−3.2 vs. −3.2	0.7834	−1.7 vs. −1.7	0.2367
Current or ex-smoker vs. never smoker	−3.2 vs. −3.7	0.0644	−1.7 vs. −1.8	0.4634
Beta blockers use, yes vs. no (baseline)	−3.2 vs. −3.1	0.4816	−1.8 vs. −1.3	**0.0095**
Calcium channel blockers, yes vs. no (baseline)	−2.9 vs. −3.4	**0.0067**	−1.7 vs. −1.6	0.7081
Long-acting nitrates, yes vs. no (baseline)	−3.4 vs. −3.1	0.0769	−2.2 vs. −1.3	**<0.0001**
Ranolazine up-titration, yes vs. no	−3.4 vs. −3.0	**0.0151**	−1.8 vs. −1.5	**<0.0001**
SBP difference, ≤5 vs. >5 mmHg	−3.2 vs. −3.2	0.9456	−1.7 vs. −1.7	0.9743
DBP difference, ≤2 vs. >2 mmHg	−3.2 vs. −3.3	0.6331	−1.7 vs. −1.6	0.6895
HR difference, ≤2 vs. >2 bpm	−3.2 vs. −3.3	0.7375	−1.7 vs. −1.5	0.0927

Abbreviations: CAD—coronary artery disease; CA—coronary angiography; PCI—percutaneous coronary intervention; CABG—coronary artery bypass grafting; CCS—Canadian Cardiovascular Society (CCS) angina class; BMI—body mass index; SBP—systolic blood pressure; DBP—diastolic blood pressure; HR—heart rate.

**Table 5:** Association between difference in angina symptoms per week and various predictors.

Angina Symptoms Difference (V1–V2) *	Coef.	*p*-Value	95% Conf.	Interval
Age	−0.0020303	0.466	−0.0074958	0.0034351
Gender	0.0223682	0.464	−0.0375502	0.0822867
BMI	−0.0140849	0.059	−0.0286865	0.0005167
Smoking status	0.−017693	0.118	−0.0393983	0.0044594
Angina symptoms per week V1	0.8766705	<0.0001	0.8534966	0.9003775
Short-acting nitrates use per week V1	0.0366705	0.014	0.0075503	0.0657906
SBP diff.	0.0050071	0.038	0.0002757	0.0097385
DBP diff.	−0.0098114	0.010	−0.0173107	−0.0023121
HR diff.	0.0096407	0.002	0.0034988	0.0157826
CCS scale_V1	−0.0000208	1.000	−0.09227	0.0922284
_cons	0.5458314	0.077	−0.0587543	1.150417

* The absolute change from visit 1 to visit 2. Abbreviations: BMI—body mass index; SBP—systolic blood pressure; DBP—diastolic blood pressure; HR—heart rate.

## Data Availability

Data are unavailable due to privacy restrictions.

## Supplementary Materials

The following supporting information can be downloaded at: https://www.mdpi.com/article/10.3390/jcm13061672/s1, Table S1: Reasons for ranolazine initiation; Table S2: Ranolazine dosage at baseline and at follow-up visit; Table S3: Adverse events (AEs) and reasons for treatment discontinuation; Table S4: Transitions among CCS angina classes from baseline to follow-up (3-months) visit; Table S5: CCS angina class and QoL score changes between the two assessments; Figure S1: Correlation between the difference in the number of angina attacks and the use of short-acting nitrates per week between baseline and follow-up visits.

## RANGER Investigators

*Investigators from hospital clinics:* Olympios Christoforos (Thriassio General Hospital of Elefsina, Athens, Greece), Dermitzakis Alkiviadis (Venizelio General Hospital, Heraklion, Greece), Efthimiadis Ioannis (Bioclinic Thessaloniki, Thessaloniki, Greece), Gardikiotis Alexandros (NIMTS, Athens, Greece), Kakouros Stavros (Sismanogleio General Hospital, Athens, Greece), Lampropoulos Stylianos (Mamatseio General Hospital, Kozani, Greece), Barbetseas John, Maria Bonou (General Hospital “LAIKO”, Athens, Greece), and Sourgounis Angelos (424 General Military Hospital, Thessaloniki, Greece). *Investigators from private practice offices:* Aggelopoulos Georgios, Agrafiotis Ilias, Alexandridis Ilias, Alexandropoulou Aikaterini, Alexoudis Apostolos, Almpanis Giorgos, Amaslidis Alexandros, Anastasiadis Filippos, Anastasopoulos Ioannis, Andreopoulou Maria, Antonios Takis, Arvanitis Panagiotis, Asimakopoulos Christos, Askar Georgios, Athanasiadis Ioannis, Aulonitis Sokratis, Chondrokoukis Georgios, Christogiannis Zacharias, Dalampuras Panagiotis, Davos Konstantinos, Dimas Konstantinos, Dimitriadis Dimokritos, Dimopoulos Vasileios, Exarchos Petros, Faka Kuriaki, Fotopoulou Vasiliki, Fragkiadakis Charalampos, Frudas Ilias, Georgiopoulos Anastasios, Georgiopoulou Aikaterini, Giakoumis Antonis, Gkiokas Stefanos, Gkiti Eleni, Gkontopoulos Arguris, Gkotsis Konstantinos, Gkraikos Athanasios, Kagiadaki Chrusa, Kalampalikis Andreas, Kalos Aggelos, Kaltsas Thomas, Kamvrogiannis Panagiotis, Kanakaraki Marina, Kanatselos Christos, Kapetanios Konstantinos, Kapnia Euaggelia, Kara Alichairidin, Karachaliou Marilena, Karagianni Stauroula, Karalioliou Sulvia, Karanikolas Nikolaos, Kargados Giorgos, Karnaras Dimitris, Katsafados Panagiotis, Katsanakis Christos, Katsikas Christos, Katsiolis Athanasios, Kleiousis Vasilis, Kokani Eirini, Kokkinomagoulou Amalia, Konstantinidis Ilias, Konstantinou Spuridon, Konstantinou Themis, Kontopoulos Georgios, Kontoroupis Georgios, Kopsida Zeta, Korlos Georgios, Kotridis Panagiotis, Koumantzias Nikolaos, Kouris Christos, Koutras Dimitrios, Koutsakis Georgios, Koutsimanis Vasileios, Krikos Vasileios, Lagatouras Dimitrios, Lappas Georgios, Lefakis Michail, Leivaditis Ioannis, Liatas Christos, Logothetis Dimitrios, Lumperopoulos Georgios, Luropoulos Stauros, Madikas Euaggelos, Mamplekos Giorgos, Mantzalardas Euaggelos, Markaki Ioanna, Markatos Gerasimos, Markomanolaki Aikaterini, Markopoulos Anastasios, Marousis Panagiotis, Martsekis Loukas, Matziridis Anestis, Mauraganis Panagiotis, Maurepis Ioannis, Michalopoulos Dionusios, Micheloggonas Ioannis, Mpamparakos Anastasios, Mpenekos Konstantinos, Mpougiatiotis Euaggelos, Mpourazanis Ioannis, Mugias Georgios, Nalmpantis Andronikos, Nikolaidis Vasileios, Ntoumpovina Marima, Osanlou Sofia, Panagiotopoulou Eleni, Panagoulias Georgios, Panisois Kon/Nos, Papadakis Emmanouil, Papadimitriou Christos, Papadimitriou Euaggelos, Papadimitriou Georgios, Papadopoulos Achilleas, Papadopoulos Apostolos, Papadopoulos Dimitrios, Papadopoulos Georgios, Papadopoulou Maria, Papaioannou Georgios, Papalumperi Elisavet, Papamentzelopoulos Spuridon, Papanastasiou Soultana, Papanikolaou Kon/Nos, Papaoikonomou Dimitris, Papas Panagiotis, Papas Panagiotis, Papavasileiou Vasileios, Patialiakas Athanasios, Patsialas Athanasios, Paulidis Vasileios, Pechlivanidis Georgios, Petrogiannis Spuros, Petropoulos Ilias, Plochorou Varvara, Pontikakis Nikolaos, Poulikarakos Panagiotis, Pournaras Georgios, Primerakis Georgios, Prionidis Markos, Psarogiannakopoulos Panagiotis, Psarrakis Charalampos, Rammos Christos, Riga Maria, Rigas Antonios, Risgits Ali, Routsakos Damianos, Rozmarakis Euaggelos, Sakellariou Dimitrios, Samartzi Maria, Samothrakitis Andreas, Sarris Isidoros, Sassalos Konstantinos, Savvalas Dimitrios, Servetas Nikolaos, Sidiropoulos Andreas, Siliogkas Georgios, Simaiakis Antonis, Simoglou Konstantinos, Sinopoulou Panagiota, Sinos Loukas, Sklirou Vasiliki, Sofillas Kosmas, Spuropoulos Kon/Nos, Stamatiou Vasileios, Stampoulidis Kon/Nos, Stathis Ioannis, Stathopoulou Konstantina, Stauropoulos Ioannis, Stauropoulos Rafail, Stefanakis Emmanouil, Stefanakis Giorgos, Stroumpoulis Prokopis, Sumeonidis Dauid, Svolis Kon/Nos, Tachmatzidis Ioannis, Tarenidis Panagiotis, Teneketzi Eleni, Thomaidis Konstantinos, Thomas Minos, Triantafullou Athanasios, Trikalinos Nikolaos, Tsakonas Kallinikos, Tsamopoulos Ioannis, Tsevekidis Charis, Tsianakas Dimitris, Tsiantis Ioannis, Tsilis Vasileios, Tsilonis Kon/Nos, Tsiotika Theodora, Tsougkos Ilias, Tziakas Dimitrios, Tziovas Aristeidis, Tzortzis Kuriakos, Vachliotis Vasileios, Vagenas Ektoras, Vardakis Kon/Nos, Vlachou Georgia, Vogas Vasileios, Vogiatzidis Konstantinos, Voukelatou Europia, Voukelatou Maria, Vrogkistinos Konstantinos, Xenos Dionusios, Zacharia Natasa, Zachos Vasileios, Zagoridis Konstantinos, Zagoridis Konstantinos, and Ziogas Sotiris.
