# Supplementary material for: Real-World Data from the Use of Ranolazine in Patients with Stable Angina Pectoris: The RANGER Study
Source: J Clin Med. 2024 Mar 14;13(6):1672. doi: 10.3390/jcm13061672 (PMC10971156; doi:10.3390/jcm13061672)
Supplement: Supplementary file 1 [file jcm-13-01672-s001.zip › jcm-2717616-supplementary.pdf]

Supplementary A

Real-world data from the use of ranolazine in patients with stable angina pectoris: the RANGER study

Tables

Table S1. Reasons for ranolazine initiation

	Total (N=1101)	Female (N=284)	Male (N=807)
**Reasons to prescribe Ranolazine**	**n (%)**	**n (%)**	**n (%)**
**Symptoms relief**	1036 (94.1)	273 (96.1)	753 (93.3)
**Improvement in every-day life activities**	983 (89.3)	252 (88.7)	721 (89.3)
**Improvement in the oxygen supply to the heart**	659 (59.8)	168 (59.2)	483 (59.8)
**Decrease the extent of ischemia in the myocardium**	642 (58.3)	148 (52.1)	488 (60.5)
**Presence of angina pectoris symptoms despite initial treatment**	549 (49.9)	132 (46.5)	412 (51.1)
**Neutral hemodynamic effect**	548 (49.8)	133 (46.8)	410 (50.8)
**Decrease acute use of nitrates**	464 (42.1)	120 (42.2)	338 (41.8)
**Other invasive techniques (PTCA, surgery) are not feasible**	400 (36.3)	81 (28.5)	314 (38.9)
**Intolerance to other agents for angina pectoris**	193 (17.5)	53 (18.7)	136 (16.9)
**Absence of occlusive coronary artery disease**	116 (10.5)	32 (11.4)	82 (10.2)
**Other reasons**	10 (1.0)	0	10 (1.0)

Table S2. Ranolazine dosage at baseline and at follow-up visit.

	Follow-up				
**Baseline**	**375mg, bid**	**500mg, bid**	**750mg, bid**	**Termination**	**Total**
**375mg, bid**	435	505	23	7	963 (87.5%)
**500mg, bid**	-	80	26	-	106 (9.6%)
**750mg, bid**	-	2	1	-	3 (0.3%)
**Total**	435 (39.5%)	587 (53.3%)	50 (4.5%)	7 (0.7%)	1072*, **

*21 patients were receiving 1 pill per day (not in accordance with the recommendations) and not included in the table. 16 of them did not change their dosage, 13 were receiving 375mg/d and 3 were receiving 500mg/d. 5 patients changed their dosage from 375mg/d to 500mg/d. 18 unknown status.
** 5 patients were receiving 375mg/d, bid and then 3 changed it to 375mg/d qd, 1 changed it to 375mg/d tds, and the last one changed it to 375mg/d hs and not included in the table.
Abbreviations: bid, twice a day; tds, three times per day; hs, half strength.

Table S3. Adverse events (AE) and reasons for treatment discontinuation

Parameter	Total (N=1101)<br>n (%)	Causality	Outcome
**Patients reported any serious AE**	2 (0.2)		
**Cardiac system**			
**2nd degree AV block**	1 (0.1)	Probable	Resolved
**Bradychardia***	1 (0.1)	Probable	Resolved
**Nervous system**			
**Dizziness***	1 (0.1)	Probable	Resolved
**Respiratory system**			
**Pulmonary embolism**	1 (0.1)	Unrelated	Death
**Patients reported any AE[a]**	11 (1.0)		
**Gastrointestinal system**			
**Constipation**	1 (0.1)	Probable	Resolved
**Diarrhoea**	1 (0.1)	Probable	Resolved
**Nausea**	3 (0.3)	Probable	Resolved
**Constipation**	1 (0.1)	Probable	Resolved
**Vomiting**	1 (0.1)	Possible	Resolved
**General disorders**			
**Malaise**	2 (0.2)	Probable/Certain	Resolved
**Central and peripheral nervous system**			
**Balance disorder**	1 (0.1)	Probable	Resolved
**Headache**	1 (0.1)	Probable	Resolved
**Head discomfort**	1 (0.1)	Probable	Resolved
**Dizziness**	3 (0.3)	Probable/ Possible/Certain	Resolved
**Respiratory system**			
**Pulmonary embolism**	1 (0.1)	Probable	Resolved
**Dyspnoea**	1 (0.1)	Probable	Resolved
**Cardiac system**			
**Atrioventricular block**	1 (0.1)	Probable	Resolved
**Musculoskeletal system**			
**Back pain**	1 (0.1)	UnaccessIble	Resolved
	**Total (n, %)**	**Female (n, %)**	**Male (n, %)**
**Patients discontinued ranolazine**	23 (2.1)		
**Reasons for treatment discontinuation**			
**Adverse events**	7 (0.7)	2 (0.2)	6 (0.6)
**Patient's decision**	6 (0.6)	2 (0.2)	4 (0.4)
**No symptoms' improvement**	4 (0.4)	1 (0.1)	3 (0.3)

**Phycisian's decision**	2 (0.2)	0	2 (0.2)
**Normal coronary arteries post coronary angiography**	1 (0.1)	1 (0.1)	0
**Financial**	1 (0.1)	1 (0.1)	0
**Poor adherence**	1 (0.1)	0	1 (0.1)
**Mental disorders**	1 (0.1)	1 (0.1)	0

[a]A patient can have reported more than one AE. *Related to the patients diagnosed and hospitalized with 2nd degree AV block.
Abbreviations: AE – adverse events; AV – atrioventricular.

Table S4. Transitions among CCS angina classes from baseline to follow-up (3-months) visit.

	**Follow-up visit**				
**Baseline visit**	**CCS I**	**CCS II**	**CCS III**	**CCS IV**	**Total**
**CCS I**	104	16	2	0	122
**CCS II**	460	244	6	4	714
**CCS III**	99	114	15	2	230
**CCS IV**	7	18	5	2	32
**Total**	670	392	28	8	1098

Abbreviations: CCS - Canadian Cardiovascular Society angina class

Table S5. CCS angina classes and QoL scores changes between the two assessments

			**QoL**			
	**CCS**		**Investigators' assessment**		**Self-reported**	
**Colour scale**	**Number of patients**	**Percentage (%)**	**Number of patients**	**Percentage (%)**	**Number of patients**	**Percentage (%)**
+2	124	11.3	723	65.7	776	70.5
+1	579	52.6	186	16.9	144	13.1
0	365	33.2	75	06.8	81	07.3
-1	24	02.2	46	04.2	34	03.1
-2	6	00.5	71	06.4	66	06.0

Abbreviations: CCS - Canadian Cardiovascular Society angina class; QoL – Quality of Life.

Color representation of changes: Green - two or more classes improvement; Light Green – one class improvement; Yellow - no change; Light Red - one class worsening; Red - two or more classes worsening.

Figures

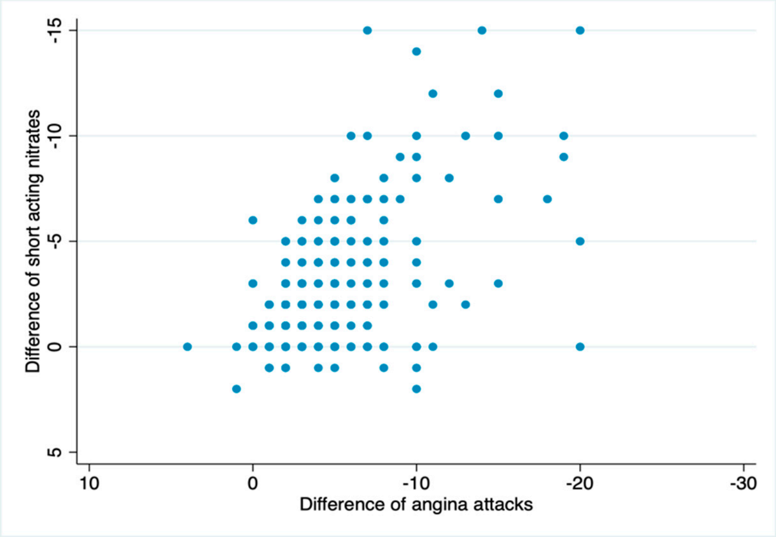

Figure S1. Correlation between the difference of angina attacks and use of short acting nitrates per week between baseline and follow-up visits.
